# Scanning Electron Microscopy Analysis of the Anterior Capsulotomy Edge: A Comparative Study between Femtosecond Laser-Assisted Capsulotomy and Manual Capsulorhexis

**DOI:** 10.1155/2018/8620150

**Published:** 2018-11-14

**Authors:** Daniele Tognetto, Chiara De Giacinto, Alberto Armando Perrotta, Tommaso Candian, Alessandro Bova, Silvia Rinaldi, Gianluca Turco

**Affiliations:** ^1^Eye Clinic, Department of Medicine, Surgery and Health Sciences, University of Trieste, Piazza Ospedale 1, 34129 Trieste, Italy; ^2^Department of Medicine, Surgery and Health Sciences, University of Trieste, Piazza Ospedale 1, 34129 Trieste, Italy

## Abstract

**Purpose:**

To compare the capsule edges ultrastructure obtained by two femtosecond laser-assisted cataract surgery (FLACS) platforms and manual continuous curvilinear capsulorhexis (CCC) using scanning electron microscopy (SEM).

**Setting:**

Eye Clinic, University of Trieste, Italy.

**Design:**

Experimental comparative study.

**Methods:**

150 anterior capsules were collected and divided into three groups as follows: Group 1 (50 capsules) obtained with manual CCC, Groups 2 and 3 (each with 50 capsules) obtained with the Catalys Laser and the LenSx Laser, respectively. All samples were imaged by means of SEM and regularity of the cut surface, and thickness of the capsule edge were evaluated and compared.

**Results:**

All femtosecond laser (FSL) capsules were perfectly circular, whereas some alteration of the circular shape was observed in the manual ones. Group 1 showed a smooth and regular capsule edge without any surface irregularity, conversely Groups 2 and 3 showed postage-stamp perforations on the capsule edge. The cut surface irregularity value in Group 2 was 1.4 ± 0.63, while it was 0.7 ± 0.49 in Group 3 (*p* < 0.05). Group 1 had a significantly lower thickness of the capsule edge than the FSL groups (*p* < 0.05). No statistically significant difference in the capsule edge thickness between the FSL groups was found (*p*=0.244).

**Conclusions:**

Despite the presence of slight cut surface irregularities, both FSL capsulotomies showed a better geometry and circularity than the manual ones. Capsulotomy specimens obtained using both FSL capsulotomies showed laser-induced alterations of the capsule edge when compared with smooth and regular edges obtained using manual CCC.

## 1. Introduction

Cataract surgery is the most commonly performed ophthalmic surgery in the world [1, 2].

Nowadays, phacoemulsification with intraocular lens (IOL) implantation is not only a treatment for visual rehabilitation but also a refractive procedure, with particular demands to achieve optimal functional results.

Anterior capsulotomy is one of the most critical and important steps in cataract surgery that may influence the correct position and centration of the IOL [3–6].

A well-centered, regular, and size-desired capsulotomy is particularly important when implanting advanced technology IOL, such as multifocal and toric IOLs, which requires a precise centration in order to maximize the IOL performance [7].

Moreover, although new IOL designs have diminished the incidence of posterior capsular opacification, a precise anterior capsulotomy is crucial in preventing the migration of lens epithelial cells [8, 9].

The most frequently used technique to obtain an anterior capsulotomy is the manual continuous curvilinear capsulorhexis (CCC) first described by Gimbel and Neuhann [10]. This technique requires special attention and surgical skills to complete it successfully and depends entirely on the experience and precision of the surgeon. Therefore, alternative options to manual CCC including the radiofrequency diathermy capsulotomy, the Fugo plasma blade, and the Zepto precision pulse capsulotomy have been described [11–13].

Currently, the introduction of femtosecond laser-assisted cataract surgery (FLACS) has changed the way of undertaking cataract surgery by automating key steps in the surgical procedure [6, 14, 15]. In particular, FLACS allows to optimize the creation of the anterior capsulotomy thus obtaining a more circular, perfect in size, and stronger aperture than manual ones [16]. In addition to the great precision, femtosecond laser (FSL) capsulotomy seems to present more capsular edge strength than manual CCC [16–18].

Although consistent circularity and predictability in size have been demonstrated with all commercially available FSL platforms, irregularities on the capsulotomy cutting edge, such as laser induced perforations and postage-stamp perforations, have been reported [16, 19–21].

These irregularities, not noticeable during surgery, are probably due to several factors such as fixational eye movements, pulse laser energy, and patient interface and could lead to an increased rate of anterior capsule tears [21, 22].

The aim of this study was to compare the capsule edge ultrastructure of the anterior lens capsule specimens obtained by two commercially available FSL platforms for cataract surgery with manual CCC by means of scanning electron microscopy (SEM). Using the same energy setting for both FSL systems, we evaluated which factors might lead to achieve the capsulotomy with the best cutting edge surface.

## 2. Materials and Methods

This is an experimental comparative study, following the tenets of the Declaration of Helsinki. All patients signed an informed consent after full explanation of the procedure.

All procedures were performed by the same experienced surgeon (DT) under topical anaesthesia.

150 capsule specimens of 150 eyes with senile cataract undergoing lens removal and IOL implantation were collected for this study.

Specimens were divided into the following 3 groups:

- Group 1 (50 eyes) included anterior capsule specimens obtained after manual CCC
- Group 2 (50 eyes) included anterior capsule specimens obtained after FSL capsulotomy using the Catalys Precision Laser System (Johnson & Johnson Santa Ana, CA, USA)
- Group 3 (50 eyes) included anterior capsule specimens obtained after FSL capsulotomy using the LenSx Laser System (Alcon Fort Worth, TX, USA)

Manual CCC was performed using capsulorhexis forceps under an ophthalmic viscosurgical device (OVD).

The laser parameters included the capsule diameter, laser pulse energy, spot separation, and layer separation. Spot separation represents the distance between two adjacent spots, whereas layer separation represents the distance to the next row.

Both FSL platforms were planned to achieve a 4.9 mm diameter anterior capsulotomy centered on the pupil. The pulse energy was 4 *µ*J in both FSL groups.

According to the suggestion of the manufacturers of the two FSL platforms, in Group 2, the spot separation was 5 *µ*m, and the layer separation was 10 *µ*m, whereas in Group 3, the spot separation and layer separation was 4 *µ*m.

The two laser systems have different patient interfaces. Catalys Laser has a fluid interface (Liquid Optic Interface, LOI) without cornea applanation, while LenSx Laser has a curved interface with a soft contact lens (SoftFit) that applanated the cornea.

Once completed laser treatment, the suction was released, the patient interface was removed, and the patient was slowly undocked from the laser. Immediately, the patient was transferred under the surgical microscope (OPMI Lumera 700, Carl Zeiss, Germany) to complete the surgical procedure. The paracentesis and primary incision were opened, and an OVD was injected into the anterior chamber above the anterior capsule. Subsequently, the anterior capsule was gently removed using capsulorhexis forceps.

After anterior capsule removal, standard phacoemulsification with IOL implantation was performed in all cases.

### 2.1. Specimens Preparation

Once removed, capsule specimens were immediately placed in a sterile container filled with fixative fluid and prepared for SEM analysis.

The samples were fixed in 2.5% glutaraldehyde in sodium cacodylate buffer and then dehydrated in an ascending series of alcohol rinses.

Specimens were mounted on aluminium stubs covered with conductive double-sided carbon adhesive tape. Next, the samples were sputtered with gold (Sputter Coater K550X, Emitech, Quorum Technologies Ltd, UK) and immediately analysed by means of SEM (Quanta250 SEM, FEI, Oregon, USA) operated in secondary electron detection mode. The working distance was adjusted in order to obtain the suitable magnification, and the accelerating voltage was set to 30 kV. The thickness was assessed with ten measurements taken along the capsulorhexis. The roughness of the cut surface, which included microgrooves, surface pitting, and notches, and overall irregularity of the cut surface, was graded on a scale of 0 to 3 [19].

### 2.2. Statistical Analysis

The Shapiro–Wilk test was used to analyse the distribution of the data. The Kruskal–Wallis and Mann–Whitney tests were used to statistically compare the differences among the anterior capsule edges characteristics. Statistical analysis was performed with SPSS software (version 17.0, SPSS Inc., Chicago, IL). For all tests, *p* was considered statistically significant when <0.05.

## 3. Results

**Figure 1:**
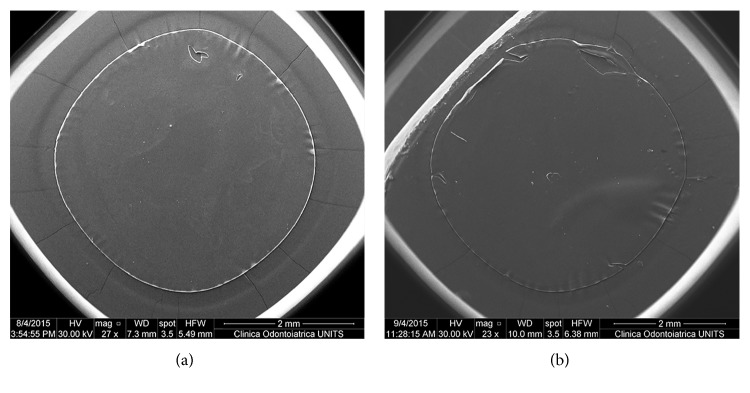
Low-magnification SEM images (original magnification 27x and 23x). Capsule samples in the FSL group show a perfect circular geometry (a). Capsule samples in the manual CCC group show some deformations such as folding and tears (b).

Low-magnification SEM images of all capsule specimens in FSL groups showed a perfectly circular geometry (Figure 1(a)), whereas some deformations such as folding and tears were observed in the manual CCC group (Figure 1(b)).

**Figure 2:**
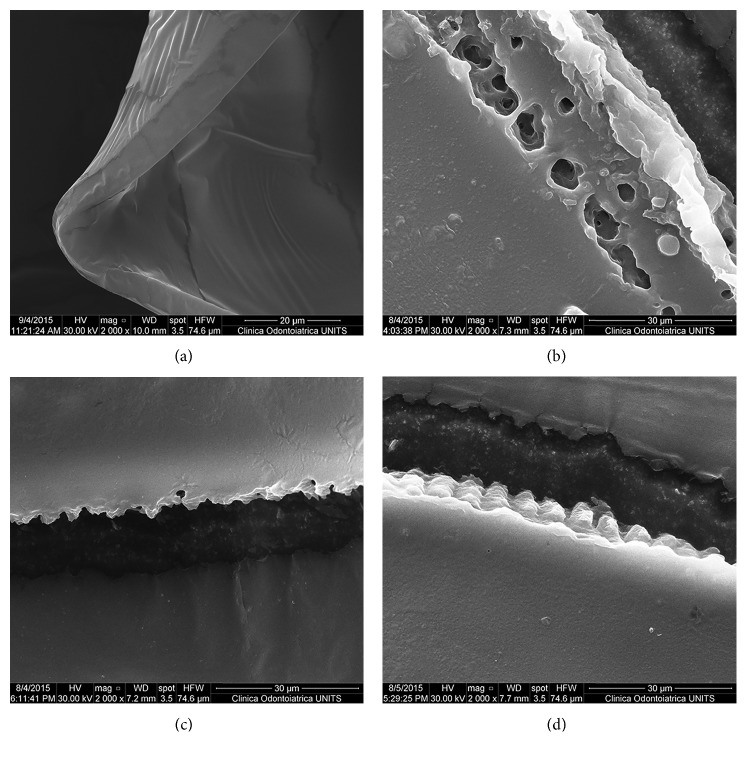
High-magnification SEM images of the capsulotomy cutting edge. SEM images show a smooth and regular surface without cut surface irregularity in the manual CCC group (a). SEM images show aberrant laser-induced perforation near the capsule edge in Group 2 (b). SEM images show postage-stamp perforations with several bumps and notches of variable width that were spread across the capsule edge in both Group 2 (c) and Group 3 (d). Original magnification: 2000x.

Regularity and thickness of the capsulotomy cutting edge was assessed at high-magnification (Table 1 and Figure 2). Group 1 showed a smooth and regular surface of the capsule edge. No cut surface irregularity was found in this group (Figure 2(a)). High-magnification SEM images revealed some differences between the FSL groups. Samples in Group 2 showed aberrant laser-induced perforations of 2.33 ± 0.44 *µ*m in size near the capsule edge (Figure 2(b)). These perforations were not found in Group 3. In both FSL groups, the regularity of the capsulotomy cutting edge was compromised by postage-stamp perforations with several bumps and notches of variable width that were spread across the capsule edge, ranging between 4 and 7 *µ*m in Group 2 (Figure 2(c)) and between 3 and 5 *µ*m in Group 3 (Figure 2(d)). The cut surface irregularity value was 1.4 ± 0.63 in Group 2 and 0.7 ± 0.49 in Group 3 (*p* < 0.05).

Group 1 had a significantly lower thickness of the capsule edge than the FSL groups (*p* < 0.05). No statistically significant difference regarding the capsule edge thickness between the FSL groups was found (*p*=0.244).

All surgeries were uneventful. No anterior capsule tears or incomplete procedures were observed.

## 4. Discussion

Nowadays, FSL technology has changed the way of undertaking cataract surgery.

This technology offers the possibility of automating crucial steps of the surgery allowing to achieve repeatable and better results than the manual technique [6, 14, 15, 23].

One of the greatest benefits in using femtosecond laser in cataract surgery is the possibility to obtain a predictable capsulotomy [16–18].

Achieving a round, well-centered, and size-desired anterior capsulorhexis is mandatory in order to obtain a perfect IOL positioning, especially with advanced technology IOL [7].

Capsulorhexis obtained with FSL had a higher circularity index than the manual technique, resulting more similar to a perfect circle [24]. Nagy et al. [14] demonstrated that capsulorhexis performed by means of FSL showed higher precision compared with the manual technique. Moreover, Friedman et al. [16] also reported that FSL capsulotomies were significantly more precise in size and shape than manual ones.

Similarly, low-magnification SEM images of our FSL capsule specimens showed a perfectly circular geometry, whereas some deformations such as folding and tears were observed in the manual CCC group.

Several studies reported irregularities of the capsule cutting edge obtained by means of FSL [16, 17, 19, 21]. In our study, the pattern of irregularity produced by both FSL platforms was similar to that described in the literature. We found a significantly high level of irregularity in all FSL samples compared with the manual ones in which no cut surface irregularity was observed. FSL specimens obtained using LenSx Laser had moderate irregularities that were spread across the capsule edge, while FSL specimens obtained using Catalys Laser had a higher level of irregularities. We did not observe anterior capsule tears in the specimens obtained with either FSL platforms as was previously reported by Abell et al. [22], whereas in concordance with their results, we found aberrant laser-induced perforations near the capsule edge in the Catalys Laser. Authors showed that these irregularities led to a significantly increased rate of anterior capsule tears [22].

Several factors might influence capsule irregularities such as laser pulse energy, laser focus, and patient interface [19–21]. Mastropasqua et al. [19] showed that capsule edge irregularities increased with increasing laser energy. Abell et al. [22] suggested that fixational eye movements could affect laser-tissue interaction resulting in anterior capsule irregularities. Ostovic et al. [25] reported that rigid curved interface FSL capsulotomy specimens showed bridges, tags, and rougher edges compared with manual CCC. Authors observed that the rigid curved interface created corneal folds and subsequently incomplete capsulotomies, while a liquid optic interface prevented incomplete capsulotomy eliminating corneal folds [25].

Several studies reported that a soft contact lens interface resulted in a smoother capsule cutting edge than a rigid curved contact lens [21, 25–27]. A correlation between edge morphology and capsule edge strength was hypothesized, suggesting that smooth regular edges are the most favorable [17].

Differently to other studies, we performed an interplatform comparison using the same energy setting for both FSL systems and retrospectively analysed which FSL features might allow to obtain the ideal edge shape. Regarding the patient interface, we observed that at the same energy setting, LenSx Laser with soft contact lens interface performed significantly smoothest capsule cutting edges than that obtained with a fluid interface of the Catalys platform. Moreover, we did not find differences in the cut surface irregularities between LenSx Laser capsulotomy and manual CCC. Our results are in concordance with results reported by Bala et al. [21] who showed that LenSx soft-fit platform was the most similar to the manual technique for fewer anomalies and homogeneity.

Other laser parameters, such as spot separation and layer separation, should be considered to better understand the microstructural differences of capsulotomy after FSL.

Studies on the Catalys Laser showed that increasing the layer separation from 10 to 15 *μ*m and using a dense spot separation result in an improvement of the cut quality and a reduction of the number of tags on the capsulotomy cutting edge [28, 29].

In our interplatform comparison, a smoothest capsulotomy cutting edge in the LenSx Laser with spot separation of 4 *μ*m compared with Catalys Laser with spot separation of 5 *μ*m was found, suggesting a better cut surface morphology using dense spot spacing.

In conclusion, the Catalys and LenSx femtosecond laser platforms allow the surgeon to perform a better capsulorhexis in geometry and circularity. As previously described, we confirm the presence of cut surface irregularities in the capsulotomies performed by means of FSL. Moreover, our study showed the smoothest capsulotomy cutting edge in the LenSx Laser if compared with Catalys Laser at the same laser energy setting, suggesting a better cut surface morphology using a soft liquid-free curved patient interface and dense spot separation. Further studies with different laser settings should be performed to find, for each commercially available FSL platform, the ideal laser parameters to achieve better surface regularity of the capsulotomy cutting edge after FLACS.

## Figures and Tables

**Table 1:** Results from the anterior capsule samples obtained from the manual technique and FSL procedure.

Group	Mean ± SD		
	Cut surface irregularity	Laser-induced perforation (*µ*m)	Thickness (*µ*m)
1	0 ± 0	0 ± 0	5.59 ± 0.32
2	1.4 ± 0.63	2.33 ± 0.44	6.39 ± 0.59
3	0.7 ± 0.49	0 ± 0	6.05 ± 0.65

## Data Availability

The data used to support the findings of this study are available from the corresponding author upon request.
